# Optimization of UAV Spraying Parameters for Pepper Pest Control Based on Droplet Deposition Characteristics and Multi-Indicator Evaluation

**DOI:** 10.3390/plants15182772

**Published:** 2026-09-10

**Authors:** Jinglei Zhang, Shuai Sun, Changfeng Shan, Guobin Wang, Cong Ma, Yubin Lan

**Affiliations:** 1 School of Intelligent Agricultural Equipment Engineering, Shandong University of Technology, Zibo 255000, China; zjl15550329775@163.com (J.Z.);; 2 Sub-Center of National Center for International Collaboration Research on Precision Agricultural Aviation Pesticide Spraying Technology, Shandong University of Technology, Zibo 255000, China; 3 Institute of Agricultural Economy and Information Technology, Ningxia Academy of Agricultural and Forestry Science, Yinchuan 750002, China

**Keywords:** plant protection UAV, pepper canopy, droplet deposition, droplet size, pest control efficacy, TOPSIS

## Abstract

Improving pesticide application efficiency in dense pepper canopies requires optimization of Unmanned Aerial Vehicle (UAV) operational parameters based on both droplet deposition and pest control performance. This study evaluated the effects of flight height and nominal droplet size setting on canopy deposition characteristics and pest control efficacy using a DJI T60 plant protection UAV under field conditions. Three flight heights (2, 3, and 4 m) and five nominal droplet size settings (100, 150, 200, 250, and 300 µm) were investigated. Droplet coverage, deposition density, canopy penetration rate, deposition uniformity, and corrected control efficacy were used as evaluation indicators, and TOPSIS was applied for comprehensive parameter optimization. The results showed that both flight height and nominal droplet size setting exert a certain influence on droplet deposition characteristics and pest control efficacy. Under the specific conditions tested in this study, the 3 m flight height achieved favorable overall deposition performance, while the 250 µm nominal setting exhibited the highest effective coverage among the tested droplet size treatments. Pest control efficacy showed a consistent trend with droplet deposition performance, and the 3 m + 250 µm nominal setting combination achieved the highest corrected control efficacy at 7 DAT. According to the TOPSIS evaluation, the combination of 3 m flight height and 250 µm nominal droplet size setting achieved the best overall performance under the experimental conditions. Because actual airborne droplet size spectra were not independently measured, the recommendation refers to the tested operational setting rather than a measured droplet size population.

## 1. Introduction

Pepper (*Capsicum annuum* L.) is an important vegetable and cash crop widely cultivated in China and worldwide [1]. Its stable production is closely related to vegetable supply, farmers’ income, and regional agricultural development. However, pepper is susceptible to pests and diseases throughout its growth cycle, making effective crop protection essential for maintaining crop health, yield, and product quality. Common insect pests in pepper fields include aphids, whiteflies, and thrips, while diseases such as Phytophthora blight can also cause substantial production losses when environmental conditions are favorable [2]. Pest infestations can directly reduce photosynthetic activity and marketable yield and may also impair fruit quality. Therefore, timely and effective pest management is an important component of stable pepper production. During the middle and late growth stages, pepper plants usually develop a compact and stratified canopy. Dense and overlapping foliage can strongly intercept spray droplets in the upper canopy, limiting droplet penetration into the inner and lower canopy layers and reducing pesticide contact with target tissues or pests [3], thereby potentially reducing control efficacy.

Conventional manual and ground-based spraying methods remain widely used in vegetable production, but they are often associated with low operational efficiency, high labor intensity, uneven deposition, and increased pesticide exposure risks for operators. Consequently, plant protection unmanned aerial vehicles (UAVs) have been increasingly adopted because of their operational flexibility, high efficiency, reduced labor demand, and adaptability to different field scales. In addition, the rotor-induced downwash airflow generated during UAV flight can assist droplet transport into crop canopies, thereby improving deposition on target surfaces and supporting more precise pesticide application [4,5].

Spray deposition during UAV application is affected by multiple operational parameters, among which flight height and droplet size are particularly important [6,7]. UAV spraying is influenced by multiple factors, including flight speed, ambient wind speed, rotor wake airflow, initial droplet velocity, and particle size distribution. Rather than treating all these factors as independent variables, this study employs a control variable approach by maintaining constant values for flight speed, spray rate, and spray width, with a focus on examining the impact of flight altitude and nominal droplet size settings on deposition within the pepper canopy. This approach aims to reduce the number of experimental factors, enabling the independent comparison of the primary effects of flight altitude and droplet size. Flight height determines the nozzle-to-canopy distance and influences the interaction between rotor downwash and crop leaves. An excessively low flight height may cause strong canopy disturbance and uneven local deposition, whereas an excessively high flight height can increase droplet dispersion, evaporation, and off-target drift. Droplet size also involves a trade-off: fine droplets generally produce higher numerical density and better spatial distribution but are more susceptible to drift and evaporation, while larger droplets show stronger drift resistance but may reduce effective coverage and canopy penetration [8,9]. Therefore, optimizing flight height and droplet size is critical for improving UAV spraying performance in pepper canopies.

Previous studies have investigated the effects of UAV operational parameters on droplet deposition in crops such as rice, wheat, maize, tea, mango, banana, and orchard canopies [10,11,12]. These studies have shown that appropriate flight parameters and droplet size settings can improve deposition quality and reduce off-target losses. However, droplet transport and interception are highly crop-specific because canopy architecture strongly influences spray redistribution. Compared with cereal crops and many orchard systems, pepper plants have a more compact and multilayered canopy, making lower-canopy deposition particularly difficult. Therefore, UAV spraying protocols developed for other crops cannot be directly transferred to pepper production without crop-specific evaluation.

In addition, UAV spray performance has often been evaluated using isolated indicators, such as droplet deposition density, coverage, or the coefficient of variation. Although these indicators are useful, single-indicator evaluation may lead to fragmented or even conflicting conclusions. For example, fine droplets may improve droplet density and uniformity, whereas intermediate droplets may achieve better effective coverage. Similarly, a given flight height may enhance upper-canopy retention but limit lower-canopy penetration. Therefore, a multi-indicator evaluation framework is needed to integrate deposition density, effective coverage, canopy penetration, and spatial uniformity. Multi-criteria decision-making methods, such as the Technique for Order Preference by Similarity to an Ideal Solution (TOPSIS), provide a practical approach for ranking treatments based on comprehensive spraying performance [13,14]. Similar multi-criteria decision-making frameworks have also been applied to agricultural spraying drone selection and UAV technology evaluation [15,16,17].

Despite the increasing use of agricultural UAVs in vegetable production, systematic studies on droplet deposition in pepper canopies remain limited. In particular, the effects of flight height and droplet size on vertical deposition distribution, canopy penetration, and effective coverage have not been fully clarified. Moreover, the influence of canopy structural differences on intra-canopy droplet redistribution has received limited attention. These knowledge gaps restrict the development of adaptive UAV spraying recommendations for pepper production.

Pepper production in the Ningxia irrigation region is characterized by a dry continental climate, strong solar radiation, low relative humidity, and frequent light winds during the spraying season. These environmental conditions can accelerate droplet evaporation and alter the interaction between rotor downwash airflow and crop canopies. Consequently, flight height and droplet size become two of the most influential operational parameters affecting droplet transport, canopy penetration, and deposition efficiency. Although these parameters have been widely investigated in cereal crops and orchards, systematic evaluations under the climatic conditions and compact canopy architecture of pepper fields in Northwest China remain limited. Therefore, this study focused on optimizing flight height and droplet size to provide region-specific recommendations for UAV spraying in pepper production.

Therefore, this study aimed to evaluate the effects of UAV flight height, droplet size, and canopy structure on droplet deposition characteristics in pepper canopies using a DJI T60 plant protection UAV under field conditions. Structured field experiments were conducted to quantify droplet deposition density, coverage, canopy penetration, spatial uniformity, and vertical deposition profiles. A TOPSIS-based multi-indicator framework was further applied to compare the comprehensive spraying performance of different treatments. The findings are expected to provide a practical basis for optimizing UAV spraying parameters and improving pesticide application efficiency in pepper production.

## 2. Materials and Methods

### 2.1. Experimental Site and Environmental Conditions

**Figure 1:**
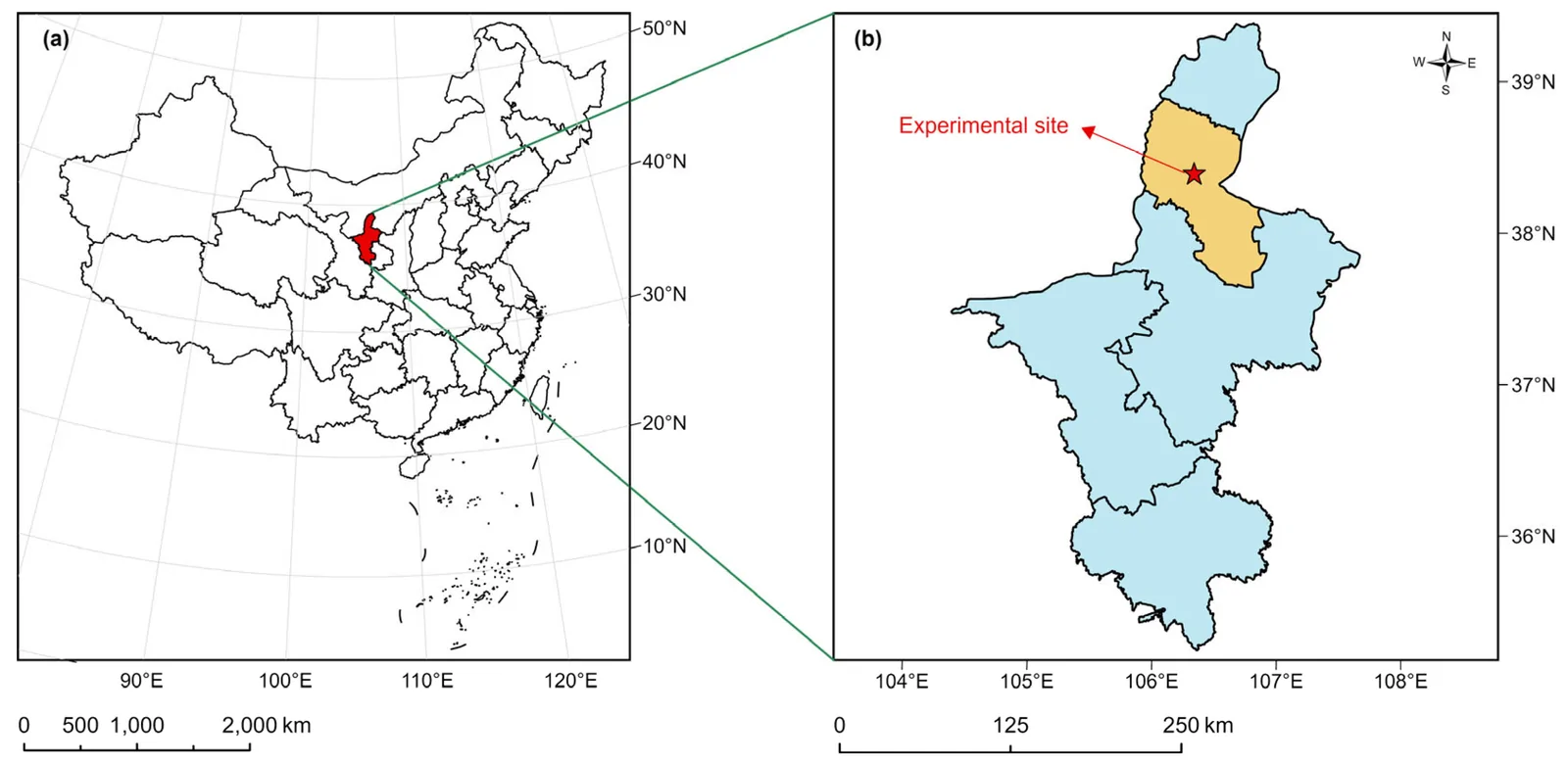
The location of the experimental site: (**a**) Ningxia Hui Autonomous Region in China; (**b**) Yinchuan City within Ningxia. The red star indicates the experimental pepper field in Yongning County.

Field experiments were conducted on 29 June 2025 in a commercial pepper field located in Yongning County, Yinchuan City, Ningxia Hui Autonomous Region, China (106°27′ E, 38°28′ N; Figure 1). The experimental field covered approximately 0.17 ha and was planted with a locally dominant commercial pepper cultivar (*Capsicum annuum* L.).

**Figure 2:**
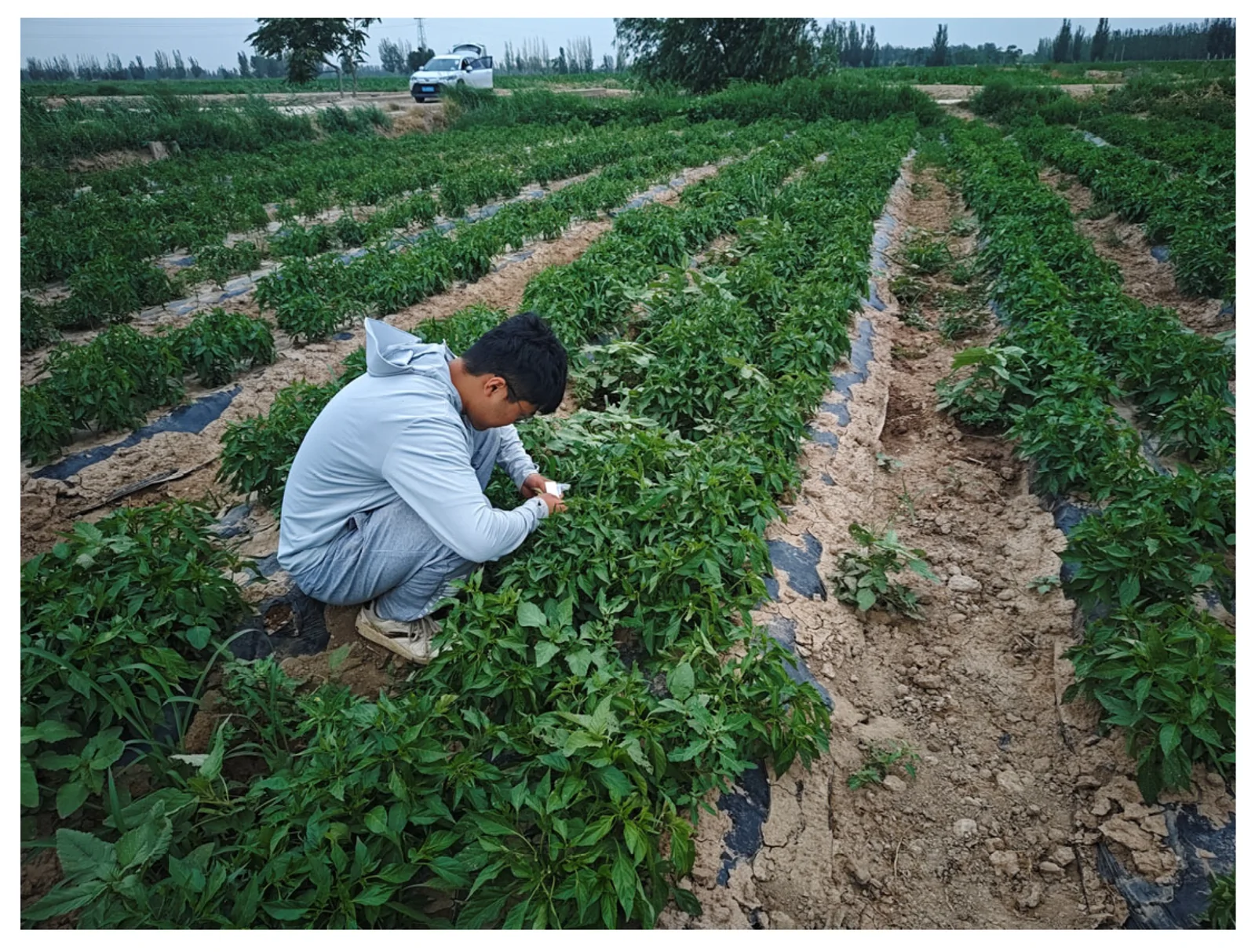
Representative growth status of pepper plants in the experimental field.

The pepper plants were arranged with a row spacing of 60 cm and a plant spacing of 30 cm (Figure 2). At the time of the experiment, the plants were at the flowering to early fruiting stage and showed relatively uniform growth within the selected experimental area. Canopy height ranged from approximately 30 to 50 cm.

During the experimental period, the ambient temperature ranged from 18.4 to 33.2 °C, with a mean temperature of 25.8 °C, and relative humidity ranged from 52.1% to 59.5%, with a mean value of 55.8%. Wind speed varied from 0 to 6.6 m/s during the monitoring period. However, to reduce the potential influence of wind-induced spray drift, UAV spraying operations were conducted only when the wind speed was below 1.0 m/s.

During spraying, the ambient wind speed was maintained below 1.0 m/s, which was substantially lower than the UAV flight speed of 6.0 m/s. Therefore, under the experimental conditions, the relative airflow associated with UAV forward motion was considerably greater than the ambient wind component. Flight speed was therefore treated as a controlled operational parameter, while ambient wind was minimized to reduce its confounding effect.

### 2.2. UAV Platform and Spraying System

**Figure 3:**
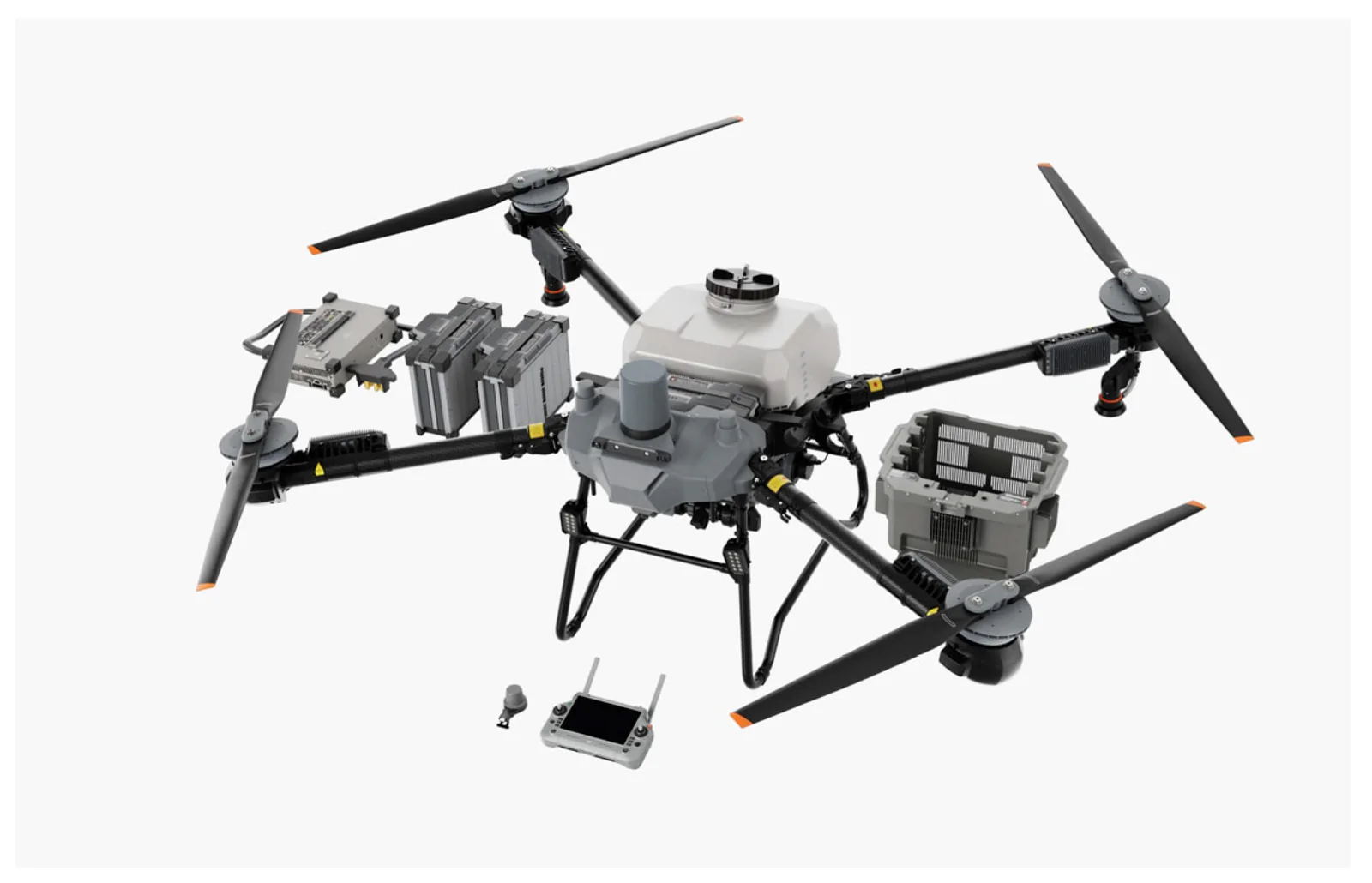
DJI T60 plant protection UAV. Note: The nozzle is located beneath the ends of the two rear rotors. The spray nozzle is located in the region affected by the downwash of the rotor, so after the droplets leave the centrifugal atomization device, they are affected by the rotor-induced airflow.

A DJI T60 plant protection UAV (DJI Agriculture, Shenzhen, China) was used for all spraying operations in this study (Figure 3). The UAV was equipped with a 50.0 L spray tank and four symmetrically mounted centrifugal nozzles. Its centrifugal atomization system allowed droplet size and spray output to be adjusted according to the experimental requirements. The main technical specifications of the UAV are summarized in Table 1.

The spraying system delivered a maximum flow rate of 18 L/min, and the atomization system allowed for nominal droplet size settings within the range of 50–500 µm. In this study, droplet size was specified according to the corresponding operational setting of the DJI T60 centrifugal atomization system. Unless otherwise stated, the reported droplet sizes (100, 150, 200, 250, and 300 µm) therefore refer to nominal operational settings rather than independently measured airborne droplet size spectra. Flight speed, effective spray swath, and application rate were kept constant unless otherwise specified. The nominal droplet size setting was adjusted through the UAV’s centrifugal atomization control system according to the manufacturer’s operating interface. Because the rotational speed corresponding to each nominal setting was not recorded during the experiment, the actual relationship between nominal setting and atomization disk rotational speed could not be quantitatively established. During field operations, the UAV used its integrated GPS navigation and radar-based terrain-following systems to maintain flight stability and a consistent spraying height above the crop canopy.

### 2.3. Experimental Design and Treatment Arrangement

A structured field experiment was designed to evaluate the effects of key UAV spraying parameters on droplet deposition within pepper canopies. To isolate the effects of individual factors before conducting the integrated performance evaluation, the experiment was organized into three comparison groups: droplet size comparison, flight height comparison, and canopy structure comparison.

For all treatments, the flight speed, effective spray swath, and spray application rate were kept constant at 6.0 m/s, 6.0 m, and 30.0 L/ha, respectively. A flight speed of 6.0 m/s was selected as a representative operational speed for the tested UAV and field conditions, while maintaining a practical balance between operational efficiency and spray application stability. Small-canopy pepper plants were used as the default target canopy unless otherwise specified. A total of eight treatments were established (Table 2). Treatment T0, corresponding to a 3.0 m flight height, a 200 µm nominal droplet size setting, and the small-canopy condition, was defined as the shared reference treatment for the three deposition comparison groups.

Using T0 as the reference treatment, the specific parameter settings for the three comparison groups were as follows. In the droplet size comparison group, the flight height was fixed at 3.0 m, and five nominal droplet size settings were evaluated: 100, 150, 200, 250, and 300 µm, with the 200 µm level corresponding to T0. In the flight height comparison group, the nominal droplet size setting was fixed at 200 µm, and three flight height levels were compared: 2.0, 3.0, and 4.0 m, with the 3.0 m level corresponding to T0. In the canopy structure comparison, T0 represented the small-canopy condition and T7 represented the large-canopy condition.

The present experimental design was not a full factorial design; therefore, the interaction between flight height and droplet size setting could not be statistically evaluated.

### 2.4. Droplet Deposition Measurement

Before the field experiments, the UAV positioning system was calibrated according to the manufacturer’s operating procedures, and the nozzle flow rates were checked to ensure stable spraying performance. Water-sensitive papers (76 mm × 26 mm, Syngenta, Basel, Switzerland) were used as droplet collectors for all treatments. To avoid cross-contamination among treatments, the spray tank was thoroughly rinsed with clean water between consecutive treatments.

**Figure 4:**
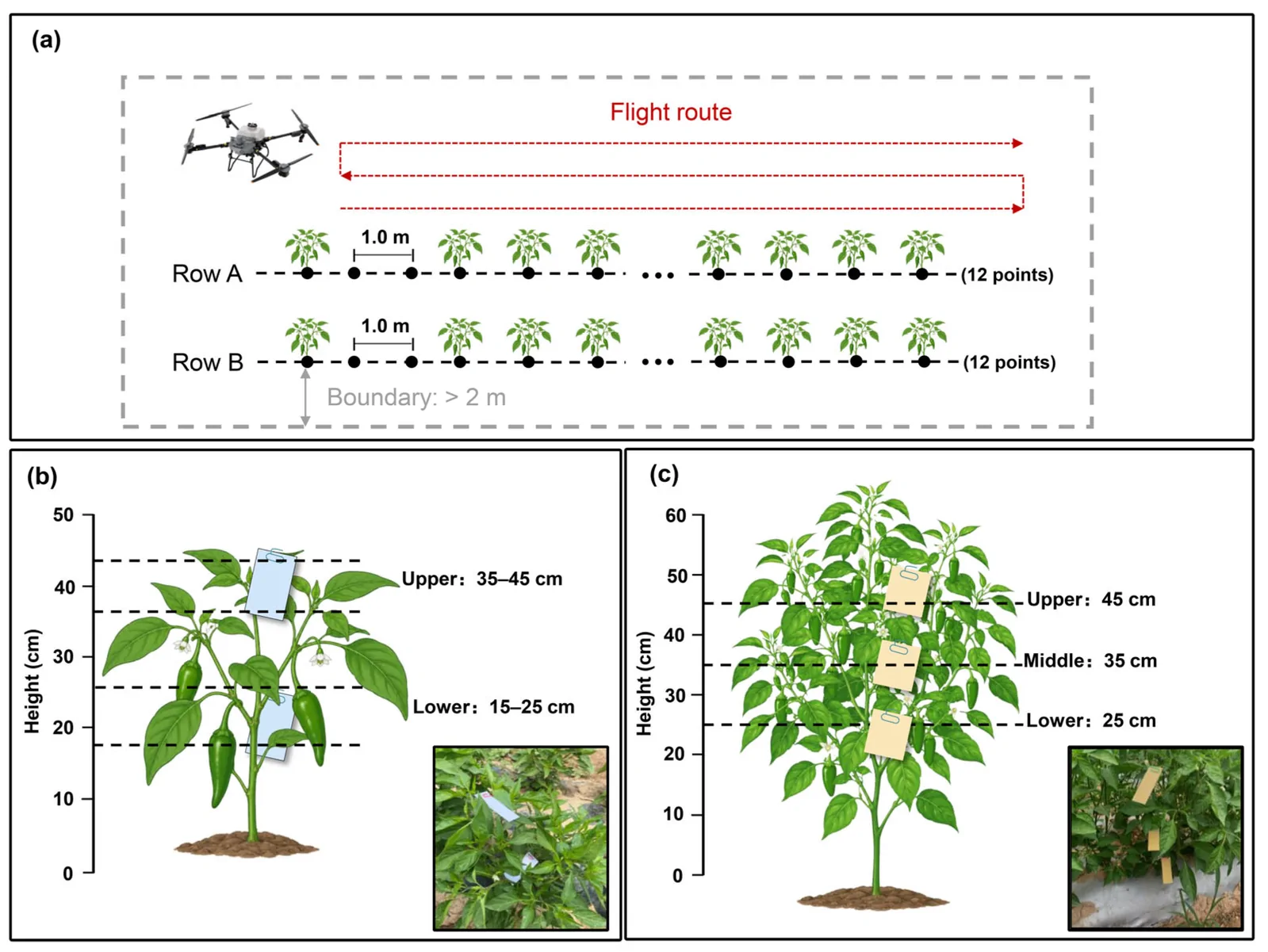
Droplet Deposition Measurement Schematic. (**a**) Field sampling layout and UAV flight route; (**b**) collector placement in small-canopy treatment, with sampling cards positioned in upper and lower canopy layers; (**c**) collector placement in large-canopy treatment, with sampling cards positioned in upper, middle, and lower canopy layers.

Droplet deposition was measured using water-sensitive paper placed at specific positions within the pepper canopy (Figure 4). All water-sensitive papers were attached to pepper leaves rather than positioned horizontally. The orientation of each collector was aligned as closely as possible with the natural orientation of the supporting leaf surface. Although this arrangement reduced the potential bias associated with horizontally positioned collectors, differences between the rigid water-sensitive papers and natural leaf surfaces may still affect droplet interception under UAV downwash. Therefore, the measured deposition values should be interpreted as collector-based estimates of canopy deposition rather than exact pesticide retention on leaf surfaces. Each treatment was conducted with three independent replicates. In each replicate, two crop rows were selected, and 12 sampling positions were arranged at 1.0 m intervals in each row, resulting in 24 sampling positions per treatment replicate. Thus, each treatment included three independent replicate spray applications, with 24 sampling positions in each replicate. All sampling positions were located at least 2.0 m away from the field boundaries to reduce potential border effects.

Collector placement differed according to canopy structure. For the small-canopy treatments, water-sensitive paper was fixed in two canopy layers: the upper canopy layer, approximately 35–45 cm above the ground, and the lower canopy layer, approximately 15–25 cm above the ground. For the large-canopy treatment, water-sensitive paper was fixed in three canopy layers, the upper, middle, and lower canopy layers, at approximately 45, 35, and 25 cm above the ground, respectively. All sampling cards were secured with paper clips to prevent displacement caused by UAV downwash airflow.

Spraying operations were conducted sequentially according to the treatment arrangement described in Section 2.3. After each application, the sampling cards were left in the field for approximately 30 min to allow the deposited droplets or stains to dry. They were then carefully retrieved and immediately sealed in labeled plastic bags according to treatment, replicate, canopy layer, and sampling position to preserve sample integrity for subsequent image and deposition analysis.

### 2.5. Image Acquisition and Parameter Extraction

**Figure 5:**
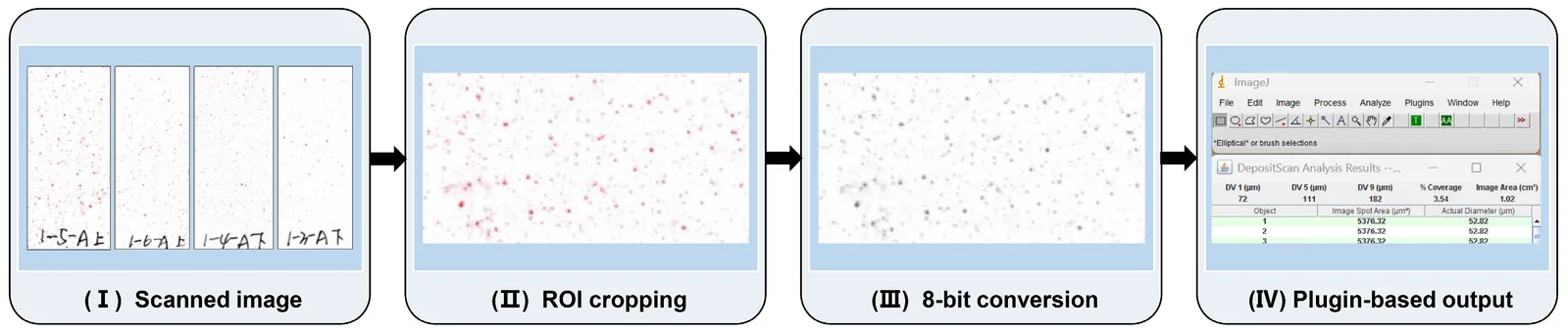
Workflow for image acquisition and droplet deposition parameter extraction.

To ensure consistency and reproducibility, droplet deposition analysis was standardized into a simple image-processing workflow (Figure 5). After collection, all sampling cards were digitized at a resolution of 600 dpi using a CanoScan LiDE300 flatbed scanner (Canon Inc., Tokyo, Japan). The scanned images were then imported into ImageJ 1.54p software (National Institutes of Health, Bethesda, MD, USA), where the effective sampling area of each collector was first cropped as the region of interest (ROI). The cropped ROI images were subsequently converted to 8-bit format for analysis.

Droplet deposition parameters were then extracted using the DepositScan plugin in ImageJ. The same image processing procedure was applied to all sampling cards to ensure consistency among samples. Through this workflow, two primary deposition parameters were obtained: droplet deposition density (*D*) and droplet coverage (*C*).

Droplet deposition density was defined as the number of detected droplet deposits per unit sampling area:

(1) $$D\;=\;\frac{N}{A_{t}}$$

where *D* is the droplet deposition density, expressed as deposits cm^−2^; *N* is the number of detected droplet deposits; and *A_t_* is the total effective sampling area (cm^2^).

Droplet coverage was defined as the percentage of the stained area relative to the total effective sampling area:

(2) $$C\;=\;\frac{A_{s}}{A_{t}}\;\times \;100\%$$

where *C* is the droplet coverage (%), *A_s_* is the cumulative droplet-stained area (cm^2^), and *A*_t_ is the total effective sampling area (cm^2^). The extracted data were then exported and organized according to treatment, canopy layer, and sampling position for subsequent statistical analysis and visualization.

Droplet deposition density and coverage describe different aspects of deposition. Deposition density represents the number of discrete droplet deposits per unit area, whereas coverage represents the fraction of the collector surface occupied by deposited stains. Therefore, a higher number of small droplets does not necessarily result in a proportionally higher coverage because coverage is also influenced by droplet diameter, spreading, coalescence, and stain size after impact. For approximately spherical droplets, the volume is related to the cube of the diameter, while the stained area on the water-sensitive paper after deposition is closer to the two-dimensional projected area or extended area. Therefore, there is no simple linear relationship between these two quantities.

### 2.6. Evaluation Indicators for Droplet Deposition

To comprehensively evaluate UAV spraying performance in pepper canopies, the extracted droplet deposition data were further quantified using spatial deposition uniformity, canopy penetration ratio, and relative vertical deposition proportion. These indicators were calculated separately according to treatment, canopy layer, and deposition characteristic.

Spatial deposition uniformity among sampling points was evaluated using the coefficient of variation (*CV*). For each treatment and canopy layer, *CV* was calculated based on droplet deposition density or droplet coverage as follows:

(3) $$CV=\frac{S}{\bar{X}}\;\times 100\%$$

(4) $$S=\sqrt{\sum_{i=1}^{n}{\left(X_{i}-\bar{X}\right)}^{2}/\left(n-1\right)}$$

where *S* is the standard deviation, *X_i_* is the droplet deposition density or droplet coverage at the i-th sampling point, $\bar{X}$ is the corresponding mean value, and *n* is the number of sampling points. A lower *CV* indicates better spatial deposition uniformity.

To characterize droplet penetration from the upper canopy layer to the lower canopy layer in the small-canopy treatments, density-based and coverage-based penetration ratios were calculated. The density-based penetration ratio (${PR}_{D}$) was calculated as follows:

(5) $${PR}_{D}=\frac{D_{L}}{D_{U}}\times 100\%$$

where $D_{L}$ and $D_{U}$ are the mean droplet deposition densities in the lower and upper canopy layers, respectively.

The coverage-based penetration ratio (${PR}_{C}$) was calculated as follows:

(6) $${PR}_{C}=\frac{C_{L}}{C_{U}}\times 100\%$$

where $C_{L}$ and $C_{U}$ are the mean droplet coverages in the lower and upper canopy layers, respectively. Higher ${PR}_{D}$ and ${PR}_{C}$ values indicate stronger downward transport of droplets and better canopy penetration.

For the canopy structure comparison, the same lower-to-upper coverage ratio defined in Equation (6) was additionally calculated for T0 and T7 to reduce the influence of the different number of canopy layers on the comparison of lower-canopy penetration.

For the canopy structure comparison, the large-canopy treatment included three canopy layers. Therefore, the relative deposition proportion ($P_{i}$) was calculated to describe the vertical deposition profile:

(7) $$P_{i}\;=\;\frac{X_{i}}{\sum_{j=1}^{k}X_{j}}\;\times \;100\%$$

where $P_{i}$ is the relative deposition proportion of the i-th canopy layer, $X_{i}$ is the mean droplet deposition density or droplet coverage of the i-th canopy layer, and *k* is the total number of canopy layers. In this study, k=2 for the small-canopy reference treatment and k=3 for the large-canopy treatment.

### 2.7. TOPSIS-Based Multi-Indicator Evaluation

To compare the comprehensive spraying performance of different flight height and droplet size treatments, a TOPSIS-based multi-indicator evaluation was conducted. TOPSIS, namely the Technique for Order Preference by Similarity to an Ideal Solution, is a multi-criteria decision-making method that ranks alternatives according to their relative closeness to the positive ideal solution and distance from the negative ideal solution [12,13]. In this study, TOPSIS was used as a relative ranking tool within the droplet size and flight height comparison groups. In addition, different weighting schemes were introduced to evaluate the stability of treatment rankings under different application priorities.

The weighting schemes were established according to the practical requirements of pesticide application and previous studies using multi-criteria evaluation for spray quality assessment. Effective droplet coverage was assigned a higher weight because adequate leaf surface wetting is directly related to pesticide efficacy and pest control performance. In contrast, deposition density, canopy penetration, and spatial uniformity were regarded as supporting indicators reflecting spray distribution characteristics. Similar application-oriented weighting strategies have been adopted in previous TOPSIS-based evaluations of agricultural spraying systems [12,13,14]. To reduce subjective bias, three weighting schemes were further designed to verify the robustness of the ranking results under different application priorities.

Four indicators were selected to construct the evaluation matrix: mean droplet deposition density ($D_{mean}$), mean droplet coverage ($C_{mean}$), coverage-based penetration ratio (${PR}_{C}$), and deposition uniformity index (UI). Droplet coverage was treated as the primary application-oriented indicator because it directly reflects the effective wetted area of the target canopy, whereas deposition density, canopy penetration, and deposition uniformity were used as supporting indicators.

For each treatment, the mean droplet deposition density and mean droplet coverage across the upper and lower canopy layers were calculated as follows:

(8) $$D_{mean}=\frac{D_{U}+D_{L}}{2}$$

(9) $$C_{mean}=\frac{C_{U}+C_{L}}{2}$$

where $D_{U}$ and $D_{L}$ are the mean droplet deposition densities in the upper and lower canopy layers, respectively, and $C_{U}$ and $C_{L}$ are the corresponding mean droplet coverages. The coverage-based penetration ratio (${PR}_{C}$) was calculated using Equation (6).

Because a lower *CV* indicates better deposition uniformity, the deposition uniformity index (UI) was converted from the coverage-based *CV* to obtain a positive indicator:

(10) $${UI}_{i}\;=1-\;\frac{{CV}_{i}\;-{\;CV}_{min}}{{CV}_{max}\;-{\;CV}_{min}}$$

where ${UI}_{i}$ is the deposition uniformity index of treatment i, ${CV}_{i}$ is the mean coverage CV of the upper and lower canopy layers for treatment i, and ${CV}_{min}$ and ${CV}_{max}$ are the minimum and maximum CV values within the same comparison group, respectively.

The *CV*-based uniformity index (UI) was used only as a normalized relative indicator within the present dataset. Because its calculation depends on the observed minimum and maximum *CV* values, UI should not be interpreted as an absolute measure of spray uniformity or directly compared across independent experiments.

Before TOPSIS analysis, all positive indicators were normalized to eliminate dimensional differences:

(11) $$r_{ij}\;=\;\frac{x_{ij}\;-x_{j}^{min}}{x_{j}^{max}\;-x_{j}^{min}}$$

where $r_{ij}$ is the normalized value of treatment i under indicator j, $x_{ij}$ is the original value, and $x_{j}^{min}$ and $x_{j}^{max}$ are the minimum and maximum values of indicator j within the same comparison group.

To test the stability of the TOPSIS ranking under different evaluation priorities, three application-oriented weighting schemes were established. For the four indicators ($D_{mean}$, $C_{mean}$, $PR_{C}$, and UI), the weights were assigned as follows:

- Scheme I, equal-weight scheme, 0.25, 0.25, 0.25, and 0.25;
- Scheme II, moderate coverage-prioritized scheme, 0.20, 0.40, 0.20, and 0.20;
- Scheme III, strong coverage-prioritized scheme, 0.15, 0.50, 0.15, and 0.20.

These schemes were used to examine whether the recommended treatment remained stable when effective canopy coverage was assigned increasing importance. Rather than assuming a single objectively determined weighting vector, three scenario-based weighting schemes were used to assess ranking sensitivity to the relative importance of effective coverage.

In the present study, droplet deposition density (*D*), coverage (*C*), penetration ratio (*PR*), and uniformity index (*UI*) were treated as benefit-type indicators, for which higher values indicate better spraying performance. After normalization, the weighted normalized decision matrix was obtained by multiplying each normalized indicator by its corresponding weight. The positive and negative ideal solutions were then identified, and the Euclidean distances from each treatment to these two ideal solutions were calculated according to the standard TOPSIS procedure. The positive ideal solution was defined by the maximum normalized weighted value of each indicator, whereas the negative ideal solution was defined by the corresponding minimum value. A higher TOPSIS score indicates that a treatment is closer to the overall optimal spraying performance. The relative closeness coefficient ($Q_{i}$) was calculated as follows:

(12) $$Q_{i}=\frac{d_{i}^{-}}{d_{i}^{+}+d_{i}^{-}}$$

where $d_{i}^{+}$ and $d_{i}^{-}$ are the distances from treatment i to the positive and negative ideal solutions, respectively. A higher $Q_{i}$ value indicates better comprehensive spraying performance. The large-canopy treatment was not included in the TOPSIS ranking because it had a different sampling structure with three canopy layers. It was used only for canopy structure-related vertical deposition profile analysis.

### 2.8. Pest Control Efficacy Evaluation

To further evaluate the practical application performance of the selected UAV spraying parameters, a field pest control verification experiment was conducted using parameter combinations established based on the TOPSIS evaluation results. A separate verification treatment code (V1–V5) was used in this experiment to avoid ambiguity because the verification experiment combined parameter levels from different comparison groups. V1–V3 corresponded to treatments in the original deposition experiment, whereas V4 and V5 were additional verification combinations. The detailed correspondence is provided in Table 3. The target pests including aphids, whiteflies, and thrips were selected according to the dominant insect species occurring during the pepper growing season. Before spraying, five sampling points were randomly selected within each plot, and the number of target pests was recorded. After UAV application, pest populations were investigated at 1, 3, 7, and 14 days after treatment.

At each investigation time, 20 representative plants were randomly selected from each plot, and the number of live pests on the upper and lower canopy layers was counted. The pest population reduction rate and corrected control efficacy were calculated according to Equations (13) and (14), respectively. The reduction rate describes the change in pest population within the treated plot, whereas corrected control efficacy accounts for the corresponding change in the untreated control and therefore provides the primary indicator for comparing treatment performance.

(13) $$Reduction\;rate(\%)=\frac{N_{0}-N_{t}}{N_{0}}\times 100$$

(14) $$Control\;efficacy(\%)=\frac{C_{t}-T_{t}}{C_{t}}\times 100$$

where $N_{0}$ represents the initial pest population before spraying, $N_{t}$ represents the pest population after treatment, $C_{t}$ represents the pest population in the untreated control plot after treatment, and $T_{t}$ represents the pest population in the treated plot after treatment.

Population reduction rate and corrected control efficacy were calculated using different equations and therefore should not be expected to have identical values.

The relationship between droplet deposition characteristics and corrected pest control efficacy was evaluated using Pearson correlation analysis. The analysis included only verification treatments that could be directly matched with the same parameter combinations in the original deposition experiment. Pearson correlation coefficients (r) and two-tailed *p*-values are reported in Section 3.6.

### 2.9. Statistical Analysis

Data compilation, preliminary processing, indicator calculation, and TOPSIS-related calculations were performed using Microsoft Excel 2019 (Microsoft Corp., Redmond, WA, USA). Statistical analyses were conducted using SPSS Statistics 26.0 (IBM Corp., Armonk, NY, USA).

For statistical analysis, for each canopy layer and deposition indicator, the measurements obtained from the 24 sampling positions within each independent replicate were first averaged to obtain one replicate-level mean. The three replicate-level means from the three independent spray applications were then treated as the experimental units for ANOVA (n = 3). Thus, the 24 sampling positions within each replicate were considered subsamples rather than independent replicates. Data are presented as the mean ± standard deviation (SD) of the three independent replicates.

According to the structured experimental design, statistical analyses were conducted within each comparison group. For the droplet size comparison group, one-way analysis of variance (ANOVA) was used to evaluate the effect of nominal droplet size setting on deposition characteristics at the fixed flight height of 3.0 m. For the flight height comparison group, one-way ANOVA was used to evaluate the effect of flight height on deposition characteristics at the fixed nominal droplet size setting of 200 µm. When significant differences were detected, Duncan’s multiple range test was applied for post hoc comparisons. Differences were considered statistically significant at *p* < 0.05.

For the canopy structure comparison, the vertical deposition profiles were analyzed descriptively because the small-canopy reference treatment included two canopy layers, whereas the large-canopy treatment included three canopy layers. The relative deposition proportions across the upper, middle, and lower canopy layers were used to characterize the influence of canopy architecture on droplet redistribution and penetration.

For the pest control verification experiment, one-way ANOVA was conducted separately within the flight height comparison (V1, V4, and V5) and nominal droplet size comparison (V1, V2, and V3) at each assessment date. When the overall ANOVA was significant, Duncan’s multiple range test was used for post hoc comparisons.

## 3. Results

### 3.1. Effect of Nominal Droplet Size Setting on Deposition Distribution Characteristics

**Figure 6:**
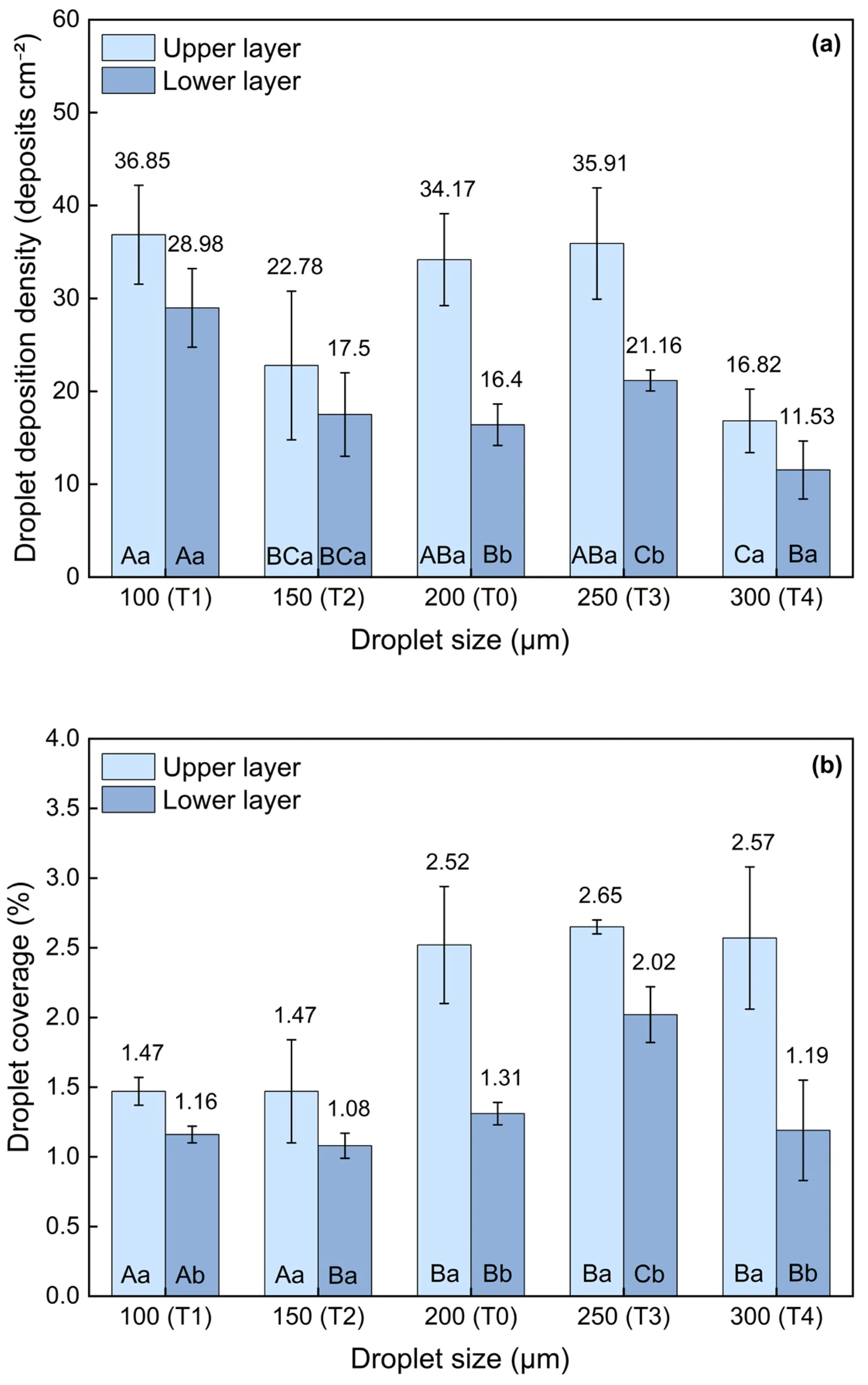
Effect of droplet size on droplet deposition density and coverage in upper and lower canopy layers: (**a**) droplet deposition density; (**b**) droplet coverage. Note: Error bars represent standard deviations. Different uppercase letters indicate significant differences among droplet size treatments within same canopy layer and deposition indicator (p<0.05). Different lowercase letters indicate significant differences between canopy layers within same droplet size treatment and deposition indicator (p<0.05).

Droplet size is a key operational parameter affecting droplet retention, coverage, and penetration in UAV spraying. Figure 6 shows the droplet deposition density and droplet coverage in the upper and lower canopy layers under different droplet size treatments, while Table 4 summarizes the spatial deposition uniformity, expressed as CV, and canopy penetration, expressed as $PR_{D}$ and $PR_{C}$.

ANOVA results showed that nominal droplet size setting had a significant effect on deposition density and coverage in the pepper canopy (p<0.05). As shown in Figure 6, droplet deposition was generally higher in the upper canopy layer than in the lower canopy layer across all droplet size treatments. Under the reference treatment T0 (200 µm), the upper canopy layer showed relatively high deposition, with a deposition density of 34.17 deposits cm^−2^ and a coverage of 2.52%. However, both indicators decreased markedly in the lower canopy layer, reaching 16.40 deposits cm^−2^ and 1.31%, respectively.

When the droplet size was reduced to 100 µm (T1), the lower canopy layer achieved the highest deposition density among all treatments, reaching 28.98 deposits cm^−2^. However, the corresponding droplet coverage remained low, with coverage values below 1.5% in both canopy layers. This indicates that although fine droplets increased the number of deposited droplets, they did not necessarily improve effective surface coverage. In contrast, the 250 µm treatment (T3) produced the highest coverage in both canopy layers. Its upper- and lower-layer coverage reached 2.65% and 2.02%, respectively, and the lower-layer coverage was significantly higher than that of the other treatments (p<0.05). These results indicate that 250 µm droplets provided a more favorable balance between droplet retention and lower-canopy deposition.

The CV results in Table 4 further showed differences in spatial deposition uniformity among droplet size treatments. For droplet deposition density, T1 showed the lowest CV values in both the upper and lower canopy layers, with values of 37.25% and 39.54%, respectively, indicating relatively uniform spatial distribution. In contrast, T0 showed higher CV values for density, with 60.23% in the upper layer and 60.33% in the lower layer. For droplet coverage, the CV values were generally higher than those of deposition density, indicating greater spatial variability in effective coverage. The coverage CV of T3 was 66.68% in the upper layer and 79.21% in the lower layer, suggesting that although T3 improved absolute coverage, spatial heterogeneity still existed within the canopy.

Canopy penetration also varied among droplet size treatments. For droplet deposition density, T1 had the highest $PR_{D}$ value of 78.64%, whereas T0 had the lowest $PR_{D}$ value of 48.00%. For droplet coverage, T1 and T3 showed relatively high $PR_{C}$ values, reaching 78.91% and 76.22%, respectively, while T0 had the lowest $PR_{C}$ value of 51.98%. These results indicate that fine droplets had an advantage in numerical penetration, whereas the 250 µm nominal setting maintained high coverage penetration while also achieving the highest absolute coverage.

Overall, droplet size caused a clear trade-off between deposition density, effective coverage, spatial uniformity, and canopy penetration. The 100 µm treatment showed advantages in deposition density, uniformity, and penetration, but its absolute coverage was relatively low. The 200 µm reference treatment showed relatively strong upper-canopy deposition but weak lower-canopy penetration. By contrast, the 250 µm treatment achieved the highest effective coverage and maintained a relatively high $PR_{C}$, indicating the most favorable overall deposition performance among the tested droplet size treatments.

Under the experimental conditions of this study, the nominal droplet size setting of 250 µm provided the best compromise among droplet coverage, canopy penetration, deposition uniformity, and drift resistance. However, the optimal droplet size is expected to vary with crop canopy architecture, meteorological conditions, UAV platform, and atomization system. Therefore, the 250 µm nominal setting was considered the preferred operational setting among those tested.

### 3.2. Effect of Flight Height on Deposition Characteristics

**Figure 7:**
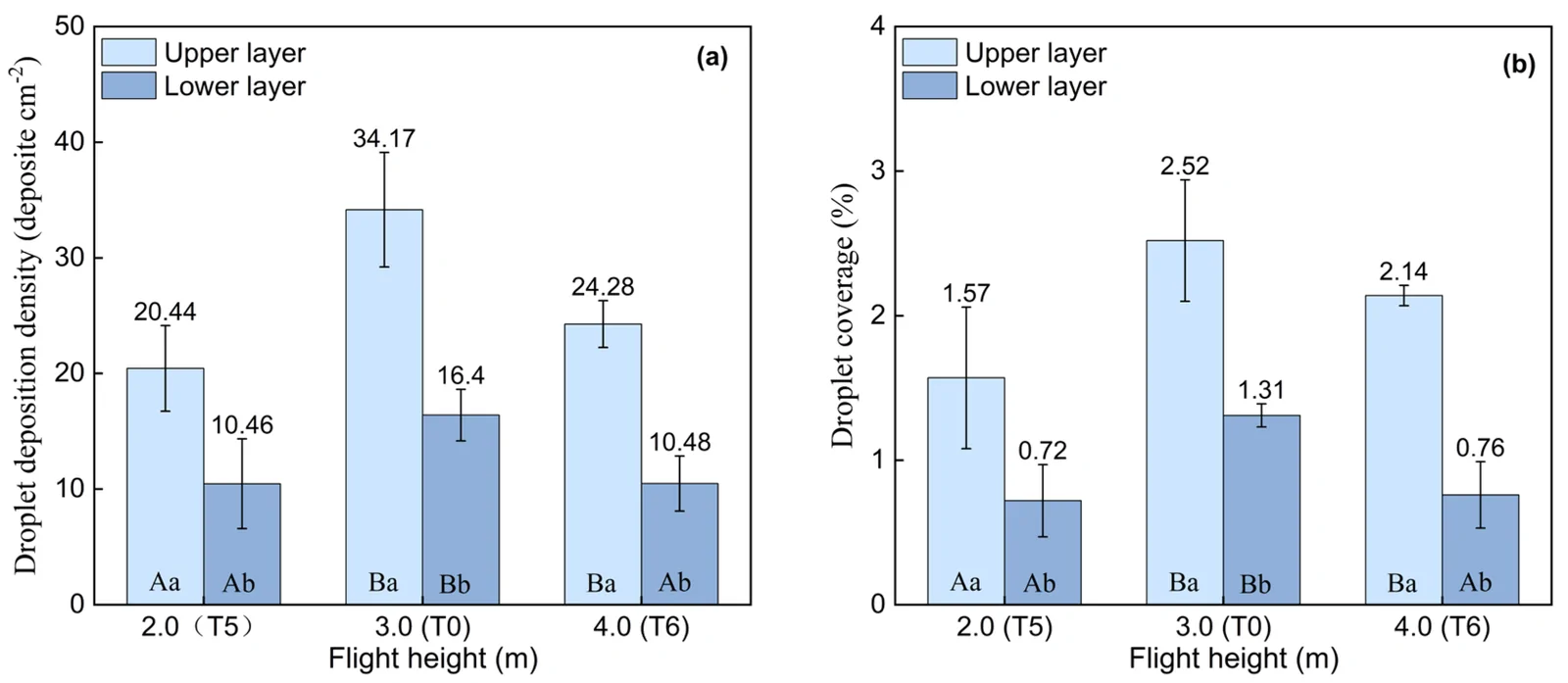
The effect of flight height on droplet deposition density and coverage in the upper and lower canopy layers: (**a**) droplet deposition density; (**b**) droplet coverage. Note: Error bars represent standard deviations. Different uppercase letters indicate significant differences among flight height treatments within the same canopy layer and deposition indicator (p<0.05). Different lowercase letters indicate significant differences between canopy layers within the same flight height treatment and deposition indicator (p<0.05).

Flight height is an important operational parameter affecting the interaction between UAV downwash airflow and the crop canopy, thereby influencing droplet transport, retention, and spatial distribution. Figure 7 shows the droplet deposition density and droplet coverage in the upper and lower canopy layers under different flight height treatments, while Table 5 summarizes the corresponding spatial deposition uniformity and canopy penetration. All treatments in this comparison were conducted at a fixed nominal droplet size setting of 200 µm.

ANOVA results showed that flight height had a significant effect on droplet deposition characteristics in the pepper canopy (p<0.05). As shown in Figure 7, both droplet deposition density and droplet coverage increased initially and then decreased as flight height increased from 2.0 to 4.0 m. The highest deposition levels were observed at 3.0 m (T0). At this height, the upper and lower canopy layers achieved droplet deposition densities of 34.17 and 16.40 deposits cm^−2^, respectively, with corresponding coverages of 2.52% and 1.31%.

When the flight height was reduced to 2.0 m (T5), deposition density and coverage decreased in both canopy layers despite the shorter distance between the UAV and the crop canopy. The upper-layer deposition density and coverage were 20.44 deposits cm^−2^ and 1.57%, respectively, while the lower-layer values decreased to 10.46 deposits cm^−2^ and 0.72%. This result suggests that an excessively low flight height may not necessarily improve effective deposition, possibly because strong near-canopy airflow can increase leaf disturbance and local spatial heterogeneity. When the flight height was increased to 4.0 m (T6), deposition also decreased compared with T0. The upper- and lower-layer coverages were 2.14% and 0.76%, respectively, indicating that excessive flight height may weaken downwash-assisted droplet transport into the lower canopy.

The CV results in Table 5 further showed that flight height affected spatial deposition uniformity. The 2.0 m treatment showed the poorest uniformity, with upper-layer CV values of 102.94% for droplet deposition density and 102.66% for droplet coverage. These high CV values indicate highly uneven deposition in the upper canopy layer under low-height spraying. In contrast, the 3.0 m treatment showed more stable spatial distribution, especially for droplet deposition density, with CV values of 60.23% and 60.33% in the upper and lower canopy layers, respectively.

Post hoc comparisons showed that the density-based penetration ratio at 2.0 m was significantly higher than that at 4.0 m, whereas the 3.0 m treatment did not differ significantly from either treatment. For coverage-based penetration, the 3.0 m treatment was significantly higher than the 4.0 m treatment, while the 2.0 m treatment did not differ significantly from either (*p* < 0.05).

Canopy penetration also varied among flight height treatments. For droplet deposition density, the highest $PR_{D}$ was observed at 2.0 m, reaching 51.20%, followed by 3.0 m and 4.0 m, with values of 48.00% and 43.18%, respectively. However, for droplet coverage, the 3.0 m treatment achieved the highest $PR_{C}$ value of 51.98%, whereas the 4.0 m treatment showed the lowest $PR_{C}$ value of 35.51%. This indicates that although the 2.0 m treatment slightly improved density-based penetration, it did not provide better effective coverage penetration.

Overall, flight height produced a clear trade-off among deposition amount, spatial uniformity, and canopy penetration. The 2.0 m treatment showed poor spatial uniformity, while the 4.0 m treatment showed insufficient lower-canopy coverage and penetration. Among the tested flight heights, 3.0 m provided the most favorable overall deposition performance, with the highest absolute deposition, relatively stable spatial distribution, and the highest coverage-based canopy penetration.

### 3.3. TOPSIS-Based Comprehensive Evaluation and Ranking Stability

The preceding single-factor analyses showed that individual deposition indicators did not always identify the same best performing treatment. In the droplet size comparison group, the 100 µm treatment (T1) showed advantages in droplet deposition density, spatial uniformity, and canopy penetration, whereas the 250 µm treatment (T3) achieved the highest effective coverage. Therefore, a TOPSIS-based comprehensive evaluation was conducted to integrate multiple deposition indicators and examine the stability of treatment rankings under different application-oriented weighting schemes.

The TOPSIS evaluation was conducted according to the indicators and weighting schemes described in Section 2.7, and the resulting relative closeness coefficients ($Q_{i}$) and rankings are presented in Table 6.

In the flight height comparison group, the 3.0 m treatment (T0) consistently ranked first under all three weighting schemes, with $Q_{i}=1.000$ in each case. This result indicates that the comprehensive advantage of the 3.0 m flight height was stable under different evaluation priorities. In contrast, the 2.0 m treatment (T5) consistently ranked last, mainly because of its lower deposition amount and poor spatial uniformity. Although the 4.0 m treatment (T6) performed slightly better than T5, its weaker lower-canopy coverage and lower $PR_{C}$ limited its overall ranking. These results indicate that, among the tested flight heights, 3.0 m provided the most favorable balance among deposition amount, spatial uniformity, and coverage-based canopy penetration.

In the droplet size comparison group, the 250 µm treatment (T3) also maintained the first-place ranking across all three weighting schemes. Under the equal-weight scheme, T3 achieved a $Q_{i}$ value of 0.656, only slightly higher than that of the 100 µm treatment (T1, $Q_{i}=0.643$). This indicates that T1 still had strong comprehensive performance when density, coverage, penetration, and uniformity were weighted equally. However, when the weight assigned to droplet coverage increased, the advantage of T3 became more evident, with $Q_{i}$ values increasing to 0.739 and 0.767 under the moderate and strong coverage-prioritized schemes, respectively. This result suggests that the recommendation of the 250 µm nominal setting is application-oriented: it is particularly suitable when effective canopy coverage is prioritized for pesticide application, rather than being superior in every individual indicator. The complete TOPSIS input matrix is provided in Supplementary Table S1 to improve the transparency and reproducibility of the comprehensive evaluation.

It is noteworthy that T0 ranked second under Scheme III despite its relatively low penetration ratio and uniformity index. This result reflects the application-oriented weighting structure rather than superiority of T0 in all individual indicators. T0 had the second-highest mean canopy coverage (1.91%) and a moderate mean deposition density, while Scheme III assigned 50% of the total weight to coverage. Consequently, its relatively favorable coverage partly compensated for its weaker penetration and uniformity in the TOPSIS distance calculation. This result illustrates that the ranking represents a weighted compromise among multiple indicators and depends on the specified application priority, rather than requiring a treatment to perform well in every individual criterion.

Overall, the TOPSIS results confirmed the ranking stability of the recommended treatments under different weighting schemes. The 3.0 m flight height remained the best-performing height treatment, while the 250 µm nominal droplet size setting consistently ranked first among the tested droplet size treatments. Considering that effective leaf surface coverage is a key requirement for practical pesticide application in dense pepper canopies, the combination of 3.0 m flight height and 250 µm nominal droplet size setting was identified as the preferred operational parameter under the tested conditions.

### 3.4. Canopy Structure-Related Vertical Deposition Profiles

**Figure 8:**
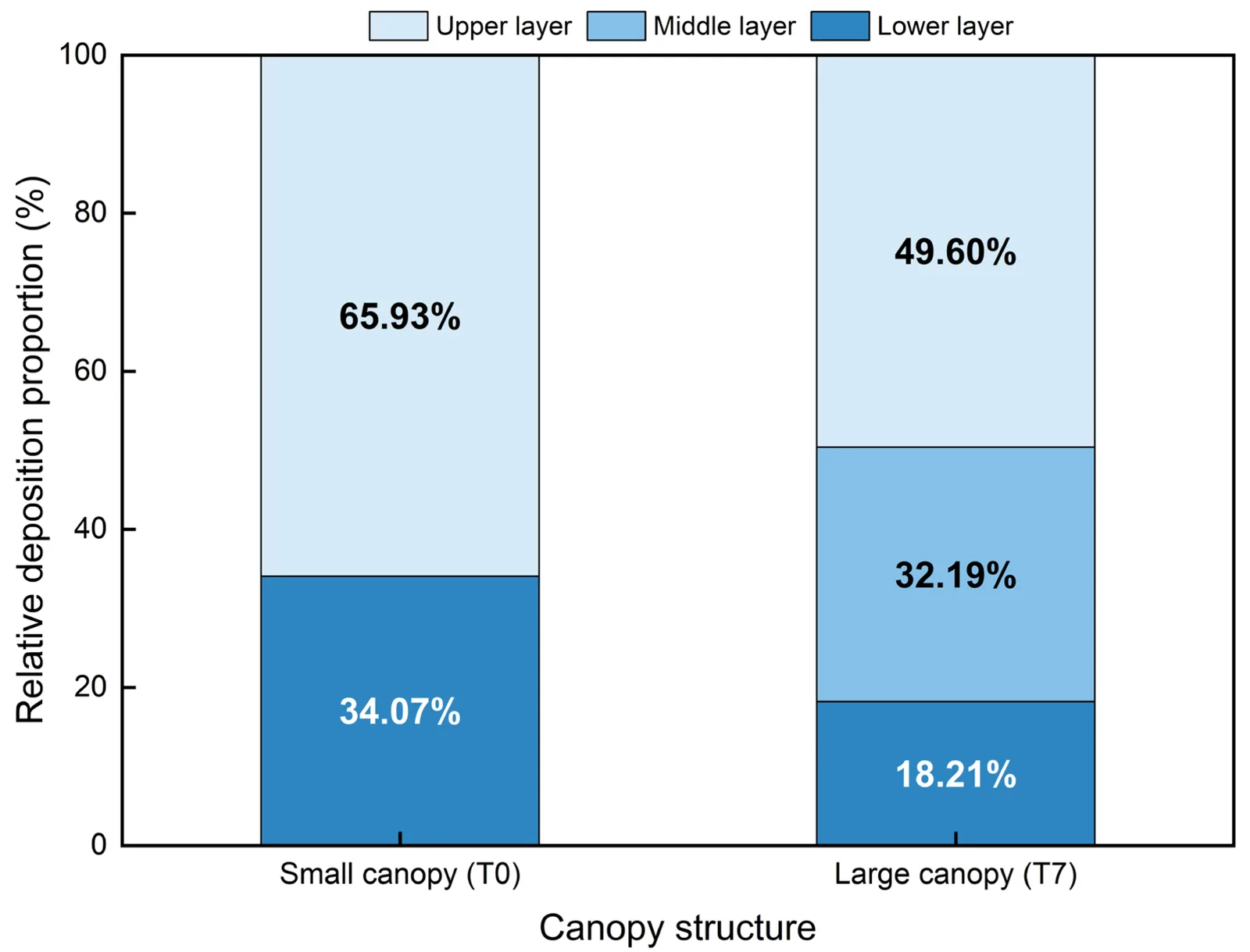
Relative vertical deposition proportions in small and large pepper canopies. Note: Relative deposition proportions were calculated based on mean droplet coverage. Small-canopy treatment T0 included only upper and lower canopy layers; therefore, no middle layer was shown.

Canopy structure affected the relative vertical distribution of droplet deposition. To evaluate this effect, the vertical deposition profiles of the small-canopy reference treatment T0 and the large-canopy treatment T7 were compared based on relative deposition proportion ($P_{i}$) (Figure 8).

In the small-canopy treatment T0, droplet deposition was distributed between the upper and lower canopy layers, accounting for 65.93% and 34.07% of the total deposition, respectively. In contrast, the large-canopy treatment T7 showed a three-layer deposition profile, with 49.60%, 32.19%, and 18.21% of deposition distributed in the upper, middle, and lower canopy layers, respectively. Compared with T0, T7 showed a lower relative deposition proportion in the lower canopy. In addition, the lower-to-upper coverage ratio was 0.52 for T0 and 0.37 for T7, indicating that the lower canopy received approximately 52% and 37% of the corresponding upper-canopy coverage, respectively. This result confirms that the lower relative deposition observed in T7 was not solely caused by the presence of the additional middle canopy layer.

These results suggest that canopy architecture plays an important role in vertical droplet redistribution. Although optimized flight height and droplet size improved overall deposition performance, lower-canopy deposition remained limited in the large-canopy treatment, highlighting the need to consider canopy structure when developing UAV spraying recommendations for pepper production.

### 3.5. Effects of UAV Spraying Parameters on Pest Control Efficacy

As shown in Table 7, the pest count data showed a clear temporal response after UAV application. Pest population reduction generally increased from 1 DAT to 7 DAT and then declined by 14 DAT. The control efficacy increased gradually after spraying and reached the maximum value at 7 days after treatment (DAT).

The higher pest suppression observed for the 3.0 m treatment was consistent with its stronger deposition performance, particularly in terms of canopy coverage, deposition density, and penetration. Because biological efficacy was not measured at every sampling position used in the deposition experiment, this consistency should be interpreted as an association between the treatment-level deposition pattern and pest control response rather than as direct proof of a causal mechanism.

The lower efficacy observed at 2.0 m and 4.0 m was accompanied by less favorable deposition patterns. The 2.0 m treatment showed relatively greater concentration of deposition in the upper canopy, whereas the 4.0 m treatment showed lower effective deposition. These observations support the importance of obtaining adequate and spatially distributed deposition within the canopy for pest control.

As shown in Table 8, corrected control efficacy followed a similar temporal pattern. The higher pest control efficacy observed for the 250 µm nominal setting was accompanied by higher effective canopy coverage. This result suggests that effective wetted area may be more relevant to biological performance than droplet number alone. The interpretation is consistent with the deposition results, in which the 100 µm nominal setting produced relatively high deposition density and penetration but comparatively lower effective coverage. Because the actual airborne droplet size spectra were not independently measured, the present result should be interpreted as an effect of the nominal atomization setting rather than a direct effect of a measured 250 µm droplet population.

Pre-treatment pest populations ranged from 33.67 ± 6.66 to 48.33 ± 9.50 among the verification treatments and untreated control. One-way ANOVA showed no significant differences in initial pest populations among the six groups (F(5, 12) = 0.570, *p* = 0.722), confirming that the plots had comparable pest pressure before UAV application.

Although V1 (3.0 m + 250 µm) consistently showed the highest mean corrected control efficacy, no significant differences in corrected control efficacy were detected among flight height or nominal droplet size verification treatments at any assessment date (*p* > 0.05).

### 3.6. Correlation Between Droplet Deposition Characteristics and Pest Control Efficacy

Pearson correlation analysis was conducted to examine the associations between droplet deposition characteristics and corrected pest control efficacy at 7 days after treatment (DAT). The analysis included the three verification treatments (V1–V3) that could be directly matched with the corresponding treatments in the original deposition experiment (n = 3 treatment-level pairs). Based on these matched treatment-level data, coverage was positively correlated with corrected control efficacy (r = 0.879, *p* = 0.316), and deposition density also showed a positive correlation with corrected control efficacy (r = 0.965, *p* = 0.168). Canopy penetration rate showed a weak positive correlation with corrected control efficacy (r = 0.268, *p* = 0.827), whereas the deposition uniformity index showed a negative correlation with corrected control efficacy (r = −0.560, *p* = 0.622). None of these correlations reached statistical significance (*p* > 0.05). Given the limited number of matched treatment combinations, these results should be interpreted as exploratory treatment-level associations rather than evidence of causal relationships.

## 4. Discussion

The present study demonstrated that UAV spraying performance in pepper canopies is jointly regulated by droplet size, flight height, and canopy structure. Under the tested field conditions, no single deposition indicator consistently identified the best treatment; rather, effective coverage, deposition density, canopy penetration, and spatial uniformity described different aspects of spray performance. This is particularly important for pepper plants because their compact and layered canopy can strongly intercept droplets before they reach the lower target surfaces. The deposition results and pest control verification further indicate that achieving adequate and spatially distributed canopy coverage is important for practical pest control.

Previous studies have shown that after leaving the nozzle, the movement speed of the droplet will rapidly decrease with the increase in the sedimentation distance, and the movement process of the droplet is jointly affected by the downwash airflow of the rotor, the flight-induced airflow and the crosswind of the environment [18,19]. Published studies provide quantitative context for these unmeasured aerodynamic variables. Zhu et al. [20] investigated the downwash airflow of a quadrotor plant-protection UAV at flight speeds ranging from 1 to 5 m/s and showed that increasing flight speed altered the downwash vortex structure and weakened airflow penetration into the lower canopy; when flight speed exceeded approximately 4 m/s, the vortex structure was less able to reach the bottom of the canopy [20]. Xue et al. [19] further reported that off-nozzle pesticide droplets lost approximately 50–80% of their velocity within 200 mm below the nozzle, and droplets smaller than approximately 150 µm were particularly sensitive to crossflow-induced drift. These literature-derived values provide quantitative physical context for the present discussion. In the present study, droplet velocity was not directly measured. Therefore, literature-reported velocity ranges are used only to provide physical context and should not be interpreted as measurements for the present experiment.

Because flight speed used in the deposition test was fixed at 6.0 m/s, the present results should be interpreted as being specific to this operational speed. A lower flight speed may increase the residence time of droplets over the canopy and potentially improve penetration in dense canopies, whereas higher speeds may reduce the effective interaction time. Future factorial experiments should therefore investigate flight speed × flight height interactions.

For the small-canopy treatments, droplet size and flight height affected deposition through different but interacting mechanisms [21]. Fine nominal settings showed advantages in deposition density and penetration, but these indicators did not necessarily translate into the highest effective coverage. This distinction is important because pest control depends on the amount and spatial distribution of pesticide reaching relevant target surfaces. The 250 µm nominal setting produced the highest effective coverage among the tested settings and also showed the highest corrected control efficacy in the verification experiment. The present data therefore support an application-oriented trade-off between droplet number, effective coverage, penetration, and uniformity. Mechanistic explanations involving drift, evaporation, downwash, or leaf disturbance are considered plausible based on previous studies [7,22,23], but were not directly measured in the present experiment and should not be interpreted as experimentally demonstrated mechanisms.

Flight height clearly affected the balance between deposition amount, spatial uniformity, and canopy penetration. The 2.0 m treatment showed greater spatial heterogeneity, whereas the 3.0 m treatment provided a more favorable balance between effective canopy deposition and penetration. At 4.0 m, lower-canopy deposition declined, indicating reduced spray delivery into the inner canopy.

Previous studies have demonstrated that UAV flight height affects the interaction between downwash airflow, droplet transport, and canopy interception [24,25,26]. The rotor-induced downwash airflow undergoes substantial vertical attenuation and redistribution as the distance below the UAV increases, and both experimental measurements and CFD simulations have confirmed that flight height strongly modifies the velocity field interacting with crop canopies [18,24]. These published aerodynamic findings provide a physical basis for interpreting the deposition differences observed in the present study. Specifically, stronger near-canopy airflow at lower flight heights may enhance canopy disturbance and promote localized droplet redistribution, whereas increasing flight height progressively weakens the effective aerodynamic interaction between the UAV downwash and the canopy, thereby reducing the transport of droplets into lower canopy layers. Because downwash velocity and leaf motion were not directly measured in the present experiment, these mechanisms should be regarded as literature-supported interpretations rather than direct experimental evidence.

More generally, droplet transport and deposition are strongly influenced by the surrounding airflow field and exposure conditions, as demonstrated in numerical studies of airborne droplet transport [27]. Similar airflow–droplet interactions may also contribute to the redistribution of UAV-generated droplets within crop canopies.

The TOPSIS evaluation further clarified why a multi-indicator framework is necessary for UAV spray optimization. Individual indicators reflected different aspects of spray performance and did not always support the same treatment. The relatively high ranking of T0 under the coverage-prioritized weighting scheme does not indicate that T0 was superior in penetration or uniformity. Rather, TOPSIS evaluates the overall distance from the ideal solution across all indicators. When greater weight was assigned to coverage, the relatively favorable coverage performance of T0 partially compensated for its weaker penetration and uniformity. Therefore, its moderate ranking reflects the weighting strategy rather than superiority in all individual indicators. For example, fine nominal droplet settings showed advantages in numerical deposition and penetration, while intermediate droplets achieved better effective coverage. In practical pesticide application, coverage is often more directly related to the contact area between active ingredients and target leaves or pests. The pest control verification showed a consistent treatment-level trend, with the 250 µm nominal setting producing the highest corrected control efficacy among the tested droplet size verification treatments. The stable TOPSIS ranking across different weighting schemes therefore provides an application-oriented basis for the recommendation, while the exploratory correlation analysis showed treatment-level trends between deposition quality and biological efficacy.

Canopy structure imposed an additional constraint on droplet redistribution. Compared with the small canopy, the large canopy had a more developed vertical structure and a greater proportion of deposition retained in the upper and middle layers, leaving a smaller proportion for the lower canopy. This observation demonstrates that canopy architecture can modify the vertical distribution of deposited spray. Similar canopy deposition constraints and application strategies have been reported in dense crop and orchard systems [28,29,30,31,32,33]. The present study did not directly measure leaf area index, leaf angle distribution, or airflow within the canopy; therefore, the observed vertical deposition pattern should be described as an empirical canopy structure effect rather than attributed to a single unmeasured mechanism. For highly developed pepper canopies, parameter optimization alone may therefore be insufficient to eliminate lower-canopy deposition deficits.

Several limitations should be acknowledged. First, this study adopted a structured comparison design rather than a full factorial design; therefore, the interaction between flight height and nominal droplet size setting was not fully quantified. Second, the droplet size treatments were based on the nominal settings of the UAV centrifugal atomization system, and the actual airborne droplet size spectra were not independently measured. Therefore, the droplet size-related results should be interpreted as the effects of nominal operational settings rather than directly measured droplet spectra. Future studies should verify Dv10, Dv50, and Dv90 under field operating conditions. Third, only three independent replicate means were available for the ANOVA of each treatment; although this design permits statistical testing, the statistical power is limited and the results should be interpreted together with variability estimates and effect sizes. Fourth, the deposition experiment used tracer-based measurements, whereas biological efficacy was evaluated in a separate pest control verification experiment. Therefore, the relationship between deposition and efficacy represents a treatment-level association rather than a direct causal test. Fifth, the experiment was conducted under a limited range of meteorological conditions and with a single UAV platform and pepper canopy development stage. Further studies should validate the recommendations under different UAV platforms, formulations, canopy structures, meteorological conditions, and pest pressures.

Because flight height and droplet size effects were evaluated in separate comparison groups, the present study cannot determine the interaction between these two factors. Therefore, the identification of 3.0 m and 250 µm as the preferred settings should be interpreted as a combination of independently optimized conditions rather than a statistically verified global optimum. A full factorial design including multiple flight heights and droplet size settings should be conducted in future studies to quantify their interaction effects.

The ultimate objective of pesticide application is effective pest suppression rather than deposition alone. In the present study, treatments with higher effective canopy coverage and favorable deposition characteristics tended to show higher corrected control efficacy. Droplet density reflects the number of discrete deposits available for potential contact with target surfaces, whereas coverage reflects the proportion of the target surface actually wetted by the spray. A high droplet density alone may therefore be insufficient if individual deposits provide limited effective coverage, while adequate coverage together with sufficient droplet density can increase the opportunity for pesticide contact with target pests or infected tissues. Canopy penetration further determines whether these deposits can reach pest habitats within the inner and lower canopy layers. Nevertheless, the correlation analysis should not be interpreted as evidence that any single deposition indicator independently determines biological efficacy.

In addition, the present study was conducted using a single UAV platform (DJI T60), and the recommended parameters may vary with rotor configuration, nozzle type, and spraying system. Because airborne droplet size spectra were not independently measured, the droplet size values reported herein should not be interpreted as measured Dv10, Dv50, or Dv90 values. The results are specific to the corresponding operational settings of the DJI T60 centrifugal atomization system. Therefore, caution should be exercised when extrapolating the present findings to other UAV platforms. Future studies should validate these recommendations using different UAV models and canopy conditions.

## 5. Conclusions

This study evaluated the effects of UAV flight height, droplet size, and canopy structure on droplet deposition in pepper canopies using a DJI T60 plant protection UAV. Under the specific conditions tested in this study, including the tested pepper cultivar, DJI T60 centrifugal atomization system, flowering-to-early-fruiting growth stage, 6.0 m/s flight speed, and wind speeds below 1.0 m/s, the results showed a clear vertical attenuation pattern, with higher droplet deposition in the upper canopy layer than in the lower layer.

Among the tested flight heights, 3.0 m provided the most favorable overall performance, balancing deposition amount, spatial uniformity, and canopy penetration. Both 2.0 m and 4.0 m were less suitable, suggesting that excessively low or high flight heights can reduce effective droplet deposition in pepper canopies.

Droplet size showed a trade-off among deposition density, coverage, penetration, and uniformity. Although 100 µm droplets performed well in density and penetration, their effective coverage was limited. In contrast, the 250 µm nominal setting achieved the highest coverage and ranked first in the TOPSIS-based comprehensive evaluation under all weighting schemes.

Canopy structure further influenced vertical droplet redistribution. The large-canopy treatment showed stronger interception in the upper and middle layers, resulting in limited lower-canopy deposition. This indicates that dense pepper canopies remain a key constraint for UAV spray penetration.

The temporal pattern of pest suppression was broadly consistent with the expected dynamics of pesticide action. The control efficacy increased rapidly after application and reached the maximum value at 7 days after treatment (DAT). Among all treatments, the 3.0 m flight height combined with the 250 µm nominal droplet size setting showed the highest control efficacy. Compared with the optimal treatment, the control efficacy decreased by approximately 9% and 17% under 2.0 m and 4.0 m flight heights, respectively. This result was consistent with droplet deposition characteristics, indicating that appropriate flight height can improve canopy penetration and pesticide utilization efficiency.

Under the experimental conditions of this study and for the DJI T60 plant protection UAV equipped with a centrifugal atomization system, a flight height of 3.0 m combined with a nominal droplet size setting of 250 µm is recommended. The optimized spraying parameters not only improved droplet deposition characteristics but also resulted in higher pest control efficacy, demonstrating the potential of UAV-based precision spraying for pepper production. These recommendations should not be directly extrapolated to other UAV platforms, cultivars, canopy structures, flight speeds, or environmental conditions without further validation. The applicability of these operational parameters to other UAV platforms or nozzle configurations should be further validated before practical adoption. Future studies should further examine height–droplet size interactions, spray drift, canopy growth stages, and pest control efficacy.

## Figures and Tables

**Table 1:** Main technical specifications of DJI T60 plant protection UAV.

Parameter	Specification
Dimensions	2870 mm × 3295 mm × 900 mm
Tank capacity	50.0 L
Spraying system	Centrifugal atomization system
Number of nozzles	4
Nozzle type	Centrifugal nozzle (LX07550SX)
Maximum spray flow rate	18 L/min
Adjustable droplet size range	50–500 µm

**Table 2:** Treatment arrangement for the structured field experiment.

Comparison Group	Treatment	Canopy Structure	Flight Height	Droplet Size
Reference treatment	T0	Small canopy	3.0 m	200 µm
Droplet size comparison	T1	Small canopy	3.0 m	100 µm
	T2	Small canopy	3.0 m	150 µm
	T3	Small canopy	3.0 m	250 µm
	T4	Small canopy	3.0 m	300 µm
Flight height comparison	T5	Small canopy	2.0 m	200 µm
	T6	Small canopy	4.0 m	200 µm
Canopy structure comparison	T7	Large canopy	3.0 m	200 µm

Note: Droplet size levels refer to the nominal operational settings of the DJI T60 centrifugal atomization system rather than independently measured airborne droplet size spectra. Throughout the manuscript, expressions such as “100 µm treatment” or “250 µm treatment” refer to the corresponding nominal operational settings.

**Table 3:** Pest Control Verification Treatment.

Verification Treatment	Flight Height	Droplet Size
V1 (original T3: 3.0 m + 250 µm)	3.0 m	250 µm
V2 (original T0: 3.0 m + 200 µm)	3.0 m	200 µm
V3 (original T4: 3.0 m + 300 µm)	3.0 m	300 µm
V4 (2.0 m + 250 µm)	2.0 m	250 µm
V5 (4.0 m + 250 µm)	4.0 m	250 µm
CK	/	/

**Table 4:** Spatial uniformity and canopy penetration under different droplet size treatments. Penetration ratios are presented as mean + SD (n = 3). Different lowercase letters indicate significant differences among droplet size treatments within same canopy layer and deposition indicator (p<0.05).

Deposition Characteristics	Droplet Size	Intra-Layer *CV* (%)		*PR* (Canopy Penetration Ratio) (%)
		Upper Layer	Lower Layer	
Droplet density	100 (T1)	37.25	39.54	78.64 ± 0.19 a
	150 (T2)	51.25	79.58	76.82 ± 3.66 a
	200 (T0, reference)	60.23	60.33	48.00 ± 0.61 c
	250 (T3)	49.67	60.77	58.93 ± 5.85 b
	300 (T4)	58.67	64.69	51.20 ± 9.96 bc
Droplet coverage	100 (T1)	51.77	61.39	78.91 ± 1.14 a
	150 (T2)	57.80	78.24	73.47 ± 4.76 ab
	200 (T0, reference)	86.98	67.52	51.98 ± 2.37 d
	250 (T3)	66.68	79.21	76.22 ± 2.61 a
	300 (T4)	73.64	62.74	68.59 ± 8.90 bc

**Table 5:** Spatial uniformity and canopy penetration under different flight height treatments. Penetration ratios are presented as mean + SD (n = 3). Different lowercase letters indicate significant differences among flight height treatments within the same canopy layer and deposition indicator (p<0.05).

Deposition Characteristics	Flight Height	Intra-Layer *CV* (%)		*PR* (Canopy Penetration Ratio) (%)
		Upper Layer	Lower Layer	
Droplet density	2 (T5)	102.94	73.92	51.20 ± 1.38 a
	3 (T0, reference)	60.23	60.33	48.00 ± 0.61 ab
	4 (T6)	67.84	70.51	43.18 ± 6.86 b
Droplet coverage	2 (T5)	102.66	92.19	45.86 ± 2.63 ab
	3 (T0, reference)	86.98	67.52	51.98 ± 8.37 a
	4 (T6)	78.39	97.69	35.51 ± 3.75 b

**Table 6:** TOPSIS relative closeness coefficients and rankings under different weighting schemes.

Comparison Group	Treatment	Scheme I $Q_{i}$	Rank	Scheme II $Q_{i}$	Rank	Scheme III $Q_{i}$	Rank
nominal droplet size setting comparison	100 µm (T1)	0.643	2	0.474	2	0.378	4
	150 µm (T2)	0.431	3	0.316	5	0.238	5
	200 µm (T0)	0.377	4	0.458	3	0.496	2
	250 µm (T3)	0.656	1	0.739	1	0.767	1
	300 µm (T4)	0.312	5	0.410	4	0.478	3
Flight height comparison	2.0 m (T5)	0.261	3	0.202	3	0.143	3
	3.0 m (T0)	1.000	1	1.000	1	1.000	1
	4.0 m (T6)	0.298	2	0.338	2	0.371	2

**Table 7:** Pre-treatment pest populations and insect population reduction rates under different verification treatments. Values are means ± SD (n = 3). Different lowercase letters indicate Duncan’s multiple range test at α = 0.05.

Comparison	Treatment	Pre-Treatment Pest Count	1 d (%)	3 d (%)	7 d (%)	14 d (%)
Flight height	V1	40.33 ± 13.01 a	46.00 ± 18.33 a	71.98 ± 7.86 a	90.11 ± 5.60 a	79.02 ± 7.46 a
	V4	45.00 ± 4.36 a	43.00 ± 13.61 a	67.99 ± 9.23 a	81.07 ± 7.10 a	73.99 ± 3.65 a
	V5	48.33 ± 9.50 a	36.00 ± 2.57 a	60.79 ± 23.29 a	73.99 ± 9.42 a	62.94 ± 14.34 a
	F	/	0.45	0.42	3.45	2.22
	P	/	0.658	0.675	0.101	0.190
Nominal droplet size setting	V1	40.33 ± 13.01 a	46.00 ± 18.33 a	71.98 ± 7.86 a	90.11 ± 5.60 a	79.02 ± 7.46 a
	V2	41.00 ± 7.00 a	41.00 ± 15.49 a	67.03 ± 8.40 a	83.96 ± 17.12 a	74.99 ± 16.69 a
	V3	47.00 ± 23.39 a	39.00 ± 6.55 a	65.01 ± 30.47 a	77.97 ± 10.12 a	68.98 ± 14.68 a
	F	/	0.19	0.11	0.78	0.42
	P	/	0.833	0.898	0.501	0.676
	CK	33.67 ± 6.66	/	/	/	/

**Table 8:** Corrected control efficacy of UAV spraying treatments at different assessment dates. Values are means ± SD (n = 3). Different lowercase letters indicate Duncan’s multiple range test at α = 0.05.

Comparison	Treatment	3 d (%)	7 d (%)	14 d (%)
Flight height	V1	77.04 ± 5.47 a	87.97 ± 9.57 a	76.93 ± 14.94 a
	V4	69.04 ± 10.61 a	78.96 ± 9.19 a	70.97 ± 13.94 a
	V5	59.03 ± 26.61 a	71.04 ± 7.87 a	60.01 ± 12.40 a
	F	0.86	2.71	1.16
	P	0.469	0.145	0.375
Nominal droplet size setting	V1	77.04 ± 5.47 a	87.97 ± 9.57 a	76.93 ± 14.94 a
	V2	72.05 ± 3.83 a	81.94 ± 18.81 a	72.09 ± 21.92 a
	V3	68.02 ± 23.52 a	74.97 ± 14.66 a	65.97 ± 20.28 a
	F	0.31	0.58	0.24
	P	0.746	0.590	0.791

## Data Availability

The original contributions presented in this study are included in the article/Supplementary Material. Further inquiries can be directed to the corresponding author.

## Supplementary Materials

The following supporting information can be downloaded at: https://www.mdpi.com/article/10.3390/plants15182772/s1, Table S1: Evaluation indicators used for TOPSIS analysis.

## Abbreviations

The following abbreviations are used in this manuscript:

UAV	Unmanned aerial vehicle
WSP	Water-sensitive paper
ROI	Region of interest
*CV*	coefficient of variation
*PR_D_*	Density-based penetration ratio
*PR_C_*	Coverage-based penetration ratio
UI	Uniformity index
TOPSIS	Technique for Order Preference by Similarity to an Ideal Solution
