# Supplementary material for: Optimization of UAV Spraying Parameters for Pepper Pest Control Based on Droplet Deposition Characteristics and Multi-Indicator Evaluation
Source: Plants (Basel). 2026 Sep 10;15(18):2772. doi: 10.3390/plants15182772 (PMC13610179; doi:10.3390/plants15182772)
Supplement: Supplementary file 1 [file plants-15-02772-s001.zip › plants-4501888-supplementary.pdf]

Table S1. Evaluation indicators used for TOPSIS analysis.

Comparison group	Treatment	D_{mean} (deposits cm^{-2})	C_{mean} (%)	PR_c (%)	*UI*
nominal droplet-size setting	T0	25.29	1.91	51.83	0.00
	T1	32.91	1.32	78.55	1.00
	T2	20.14	1.28	73.64	0.45
	T3	28.53	2.34	76.17	0.21
	T4	14.17	1.88	46.25	0.44
Flight height	T0	25.29	1.91	51.83	1.00
	T5	15.45	1.15	45.83	0.00
	T6	17.38	1.45	35.74	0.47

Note: C_{mean} and D_{mean} represent the mean droplet coverage and droplet deposition density averaged across the upper and lower canopy layers, respectively. *PRc* represents the coverage- based canopy penetration ratio. *UI* represents the deposition uniformity index converted from the mean intra-layer *CV* of droplet coverage. TOPSIS normalization was performed separately within each comparison group.
